# The third species of the genus *Pachypaederus* Fagel, 1958 (Coleoptera, Staphylinidae, Paederinae) from the Oriental region

**DOI:** 10.3897/zookeys.1037.67300

**Published:** 2021-05-11

**Authors:** Xiaoyan Li, Yanpeng Cai, Haifeng Chen

**Affiliations:** 1 Hebei Key Laboratoryof Animal Diversity, Langfang Normal University Aiminxidao 100, Anci Area, Langfang 065000, Hebei Province, China Langfang Normal University Aiminxidao 100 Langfang China; 2 Morphological Laboratory, Guizhou University of Traditional Chinese Medicine, Guiyang, 550025, Guizhou, China Guizhou University of Traditional Chinese Medicine Guiyang China

**Keywords:** China, Paederina, taxonomy, Yunnan

## Abstract

A new species of *Pachypaederus* Fagel, 1958, *P.kongshuhensis* Li, **sp. nov.**, is described from Yunnan Province, China. This species represents the third member of the genus from the Oriental region. Color plates of the habitus, labrum, mandibles, sternites VII–IX of the male and female, as well as the aedeagal structures are provided. A key to Oriental *Pachypaederus* species is provided.

## Introduction

The genus *Pachypaederus* belongs to the subtribe Paederina, with *Paederus crassus* Boheman as the type species by original designation (Fagel 1958) and another 10 species originally included in this group (Fagel 1958). The group has a limited distribution and is only documented from the Ethiopian Region (South Africa, Guinea, Cameroon, D.R. Congo, Uganda, Rwanda, Tanzania, Zambia, Zimbabwe, Madagascar) (Fagel 1958; Janák 1998; Willers 2003; Janák 2012) and the Oriental Region (Chinese provinces of Yunnan and Myanmar) (Willers 2001, 2002). So far, there are 27 species known, including 25 Ethiopian species and 2 Oriental species (Willers 2003; Janák 2012; Li et al. 2019).

Based on the original description by Fagel (1958) (large size, reduced hind wings, carinate prosternum, narrowly separate and parallel gular sutures, free and filiform parameres, robust aedeagus as in the genus *Allopaederus*), *Pachypaederus* is not well differentiated from the other genera of Paederina. Furthermore, *P.capillaris* does not have a carinate prosternum (V. Assing pers. comm.). In consideration of the morphological diagnosis, disjunct distribution between Afrotropical and Oriental regions, and preliminary phylogenetic work by Li and Zhou (2009), the monophyly of the genus *Pachypaederus* is doubtful and needs further assessment in future. Here, we follow the Catalog of Chinese Coleoptera edited by Li et al. (2019).

In 2016, some rove beetle specimens were collected from the border of Yunnan, China, and Myanmar. After close examination, some of these were discovered to belong to a new species of *Pachypaederus*. This study describes the new species and updates the information of this genus. As a result, the number of species in this genus from the Oriental Region increases to three.

## Materials and methods

The dried specimens were softened in hot water at 60 °C for about 8 hours for dissection of the abdominal terminalia. The male genital was soaked in 10% KOH solution (30 °C) for about 20–40 minutes (depending on the degree of sclerotization). The surrounding soft tissues were immediately removed, and the dissected parts were preserved in glycerin in plastic microvials with stoppers for the subsequent observation and photography. Two specimens were dissected in this study.

Observation, dissection, and measurements were done under a Zeiss SteREO Discovery V20 stereomicroscope. Photos of the habitus, sternites, and genitalia were taken with Zeiss AxioCam MRc 5 camera attached to a Zeiss Axio Zoom V16 stereo zoom microscope. Photographs were synthesized and stacked with Zen 2012 (Blue version) and Helicon Focus imaging software. Inkscape v. 0.91 was used to make the line drawings.

All specimens listed in the present study were deposited in the Institute of Zoology, Chinese Academy of Sciences (IZ-CAS).

The following abbreviations are used in the descriptions:

**AEL** aedeagus length (base of median lobe to apical part);

**AEW** aedeagus width (greatest width of pronotum);

**BL** body length (from anterior margin of labrum to end of abdomen);

**EL** elytra length (from humeral angle to posterior margin);

**ESL** elytra suture length (apex of scutellum to apex of elytral suture);

**EW** elytra width (width of elytra across the widest part);

**EYL** eye length (longitudinal length of eye in dorsal view);

**FL** forebody length (from anterior margin of labrum to posterior margin of elytra);

**HL** head length (from anterior margin of clypeal to posterior constriction of head);

**HW** head width (greatest width of head, included eyes);

**PL** pronotum length (from anterior margin of pronotum to its posterior margin);

**POL** postocular length (from posterior margin of eye to posterior constriction of head);

**PW** pronotum width (greatest width of pronotum).

## Taxonomy

### Pachypaederus

Taxon classificationAnimaliaColeopteraStaphylinidae

Fagel, 1958: 68, 70

40729B20-4F48-564D-BA4E-F5705DCE3432

#### Type species.

*Paederus crassus* Boheman, fixed by original designation.

### Key to *Pachypaederus* of the Oriental region

**Table:** 

1	Body black including antennae, pronotum and abdomen	***P.pallitarsis* Willers**
–	Body with pronotum and at least first four abdominal segments reddish brown	**2**
2	Interior armature complex, with more sclerites; apex of dorsal plate in aedeagus curved to the right in ventral (parameral) view (Figs 2A, 3A)	***P.kongshuhensis* sp. nov.**
–	Interior armature with fewer sclerites; apex of dorsal plate in aedeagus curved to the left in ventral (parameral) view (Willers 2001: 191)	***P.capillaris* Fauvel**

### Pachypaederus kongshuhensis

Taxon classificationAnimaliaColeopteraStaphylinidae

Li sp. nov.

98928FAF-C5A2-5780-BAD2-79B1DCDB54E5

http://zoobank.org/4C2A985F-8470-4D0F-BA60-9AAE5A8ADA56

Figures 1 , 2 , 3

#### Type material.

***Holotype*:** ♂, **China: Yunnan** Province: Tengchong County, Mingguang Town, Kongshuhe County (空树河村), 2100 m elev., 25.7245°N, 98.6341°E, 30.VI.2016, coll. by Xiaoyan Li (IZ-CAS). ***Paratypes***: ♂, 2 ♀♀, same data as holotype (IZ-CAS).

#### Description.

BL: 8.7–9.0 mm; FL: 3.6–3.8 mm. HL: 1.08 mm; HW: 1.25 mm; PL: 1.39 mm; PW: 1.17 mm; EL: 1.08 mm; EW: 1.39 mm; EYL: 0.36 mm; POL: 0.50 mm.

Body glossy with typical “Paederus” color pattern; head and two apical segments of abdomen, the apical half of femora, and basal two-thirds of elytra dark blue; other parts brown; antennomeres 1 and 2 and 9–12 brownish yellow. Abdominal segments 4–6 with black patches in middle, and patches decreasing in size anteriad.

Head wider than long (average HL/HW = 0.86). Eyes moderately large (average HL/EYL = 3.0), protruding laterally. Diameter of eye longer than gena and shorter than temple (average ratio, gena: eye: temple = 0.70: 1: 1.38). Surface of head smooth. Vertex and frons glabrous with sparse punctures, lateral portions of head with more denser, shallower, and coarser punctures which are irregularly distributed and of variable size.

Antennae filiform and densely pubescent, starting from antennomere 9. Ventral portion of neck with a reversed V-shaped protrusions.

Pronotum slightly longer than wide (average PL/PW = 1.12). Scutellum glossy with reticulate microsculpture and fine setiferous punctation. Mesoventrite pressed, surface smooth, with fine microsculpture, and anterior margins ridged. Metaventrite small with anterior portion even and posterior surface pressed with fine microsculpture.

Elytra trapezoidal-sided, longer than wide (average EL\EW = 0.79 and ESL/EL = 0.68), wings reduced completely. Surface uneven, lustrous, with fine reticulate microsculpture, punctures smaller and denser than that on pronotum, diameter of a puncture usually shorter than interval between punctures.

Tergites III–VI of abdomen with setiferous punctures small and sparse; basal area of each tergite without distinct punctate row. Sternites with punctation similar to that on tergites.

**Figure 1.**
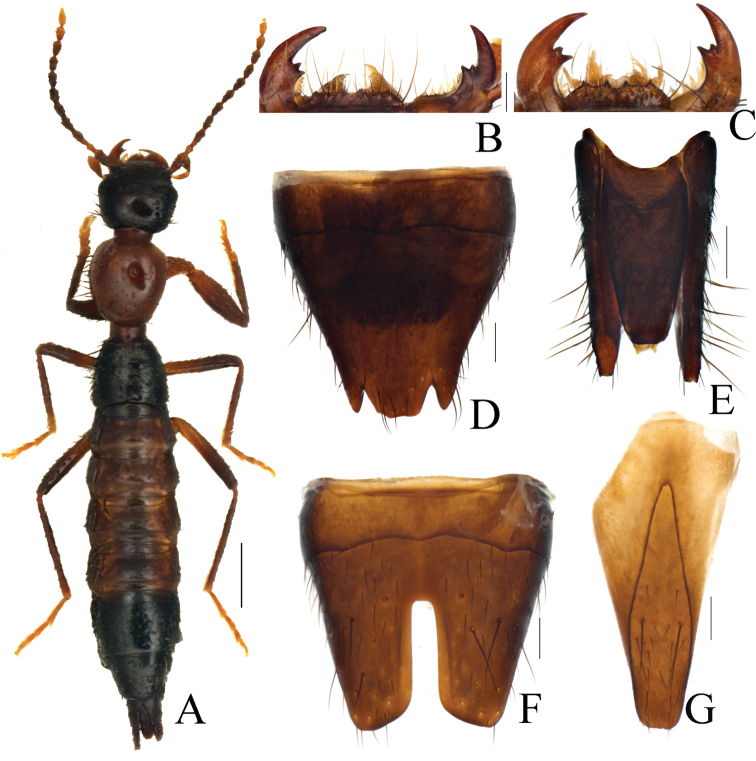
*Pachypaederuskongshuhensis* Li, sp. nov., morphology **A** habitus **B** labrum and mandibles in male **C** labrum and mandibles in female **D** sternite VIII in female **E** sternite IX and lateral proctiger in female **F** sternite VIII in male **G** sternite IX in male. Scale bars: 1.0 mm (**A**); 0.2 mm (**B–G**).

**Male.** Labrum (Fig. 1B) narrower than in female, with two small pairs of teeth on anterior margin. Tergite VIII with punctures denser and larger than those on tergites III–VII. Sternites III–VII with setae slightly directed mediad. Sternite VII with microsculpture and punctures much denser than those on sternites III–VI, posterior margin broadly notched. Sternite VIII with deep, parallel-sided, median incision, base of incision truncate, depth longer than half the length of sternite (Fig. 1F). Sternite IX asymmetrical, apex slightly emarginate and inner ridge symmetrical (Fig. 1G).

**Female.** Labrum (Fig. 1C) well developed, distinctly wider than that in male, two pairs of anterior teeth distinctly larger and sharper. Sternite VIII deeply notched as in Figure 1D. Sternite IX (Fig. 1E) with basal part bilobed and gradually narrowed apically, posterior margin shallowly emarginated in middle.

**Figure 2.**
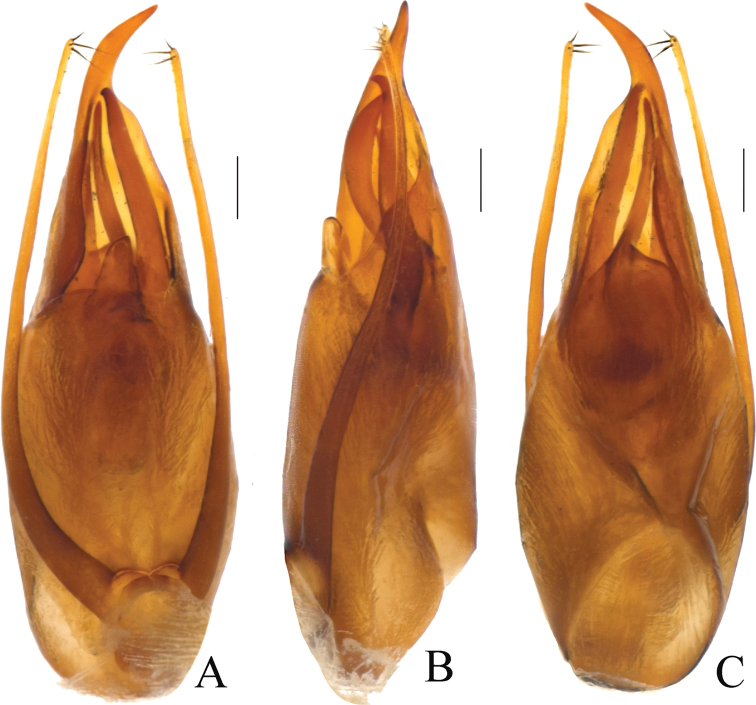
*Pachypaederuskongshuhensis* Li, sp. nov., morphology **A** aedeagus, in ventral view **B** aedeagus, in lateral view **C** aedeagus, in dorsal view. Scale bars: 0.2 mm.

**Figure 3.**
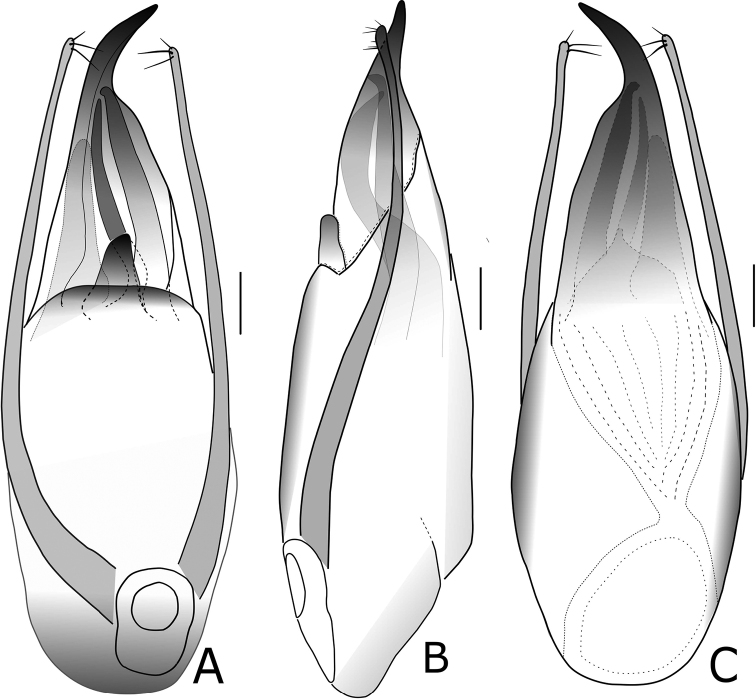
*Pachypaederuskongshuhensis* Li, sp. nov., morphology **A** aedeagus, in ventral view **B** aedeagus, in lateral view **C** aedeagus, in dorsal view. Scale bars: 0.2 mm.

Aedeagus (Figs 2A–C, 3A–C), robust and asymmetrical. AEL = 2.27 mm, AEW = 0.5 mm. Parameres symmetrical and filiform in shape; posterior part slightly clavate. Dorsal plate long and asymmetrical, gradually narrower posteriad and apical part rather sharp and curved leftward in dorsal view. Ventral plate short, with apex broadly round. Internal sac complex and exposed in ventral side, the interior armature with several elongate sclerites and several broad and lamelliform sclerites.

#### Diagnosis.

The new species *Pachypaederuskongshuhensis* sp. nov. can be easily distinguished from the two Oriental congeners by a combination of bicolored body and the morphology of the aedeagus. *Pachypaederuspallitarsis* is completely black, while in *P.capillaris*, the apex of the dorsal plate faces to the left and there are fewer sclerites in the internal sac of the aedeagus.

#### Distribution.

The species is known only from westernmost Yunnan Province, China, at an altitude of 2100 m. The specimens were collected in June as they were moving under fresh grass along the sides of a river.

#### Etymology.

The specific epithet is derived from the type locality, Kongshuhe, a mountain village in westernmost Yunnan Province, China, near the border between Myanmar and China.

## Supplementary Material

XML Treatment for Pachypaederus

XML Treatment for Pachypaederus kongshuhensis
